# Anaemia in women of reproductive age in low- and middle-income countries: progress towards the 2025 global nutrition target

**DOI:** 10.2471/BLT.20.280180

**Published:** 2022-01-20

**Authors:** Md Mehedi Hasan, Ricardo J Soares Magalhaes, Sarah P Garnett, Yaqoot Fatima, Md Tariqujjaman, Sonia Pervin, Saifuddin Ahmed, Abdullah A Mamun

**Affiliations:** a Institute for Social Science Research, The University of Queensland, 80 Meiers Road, Long Pocket Precinct, Indooroopilly, Queensland 4068, Australia.; b Spatial Epidemiology Laboratory, School of Veterinary Science, The University of Queensland, Gatton, Australia.; c Children's Hospital Westmead Clinical School, The University of Sydney, Westmead, Australia.; d Nutrition and Clinical Services Division, International Centre for Diarrhoeal Disease Research Bangladesh (icddr,b), Dhaka, Bangladesh.; e Population, Family and Reproductive Health, Johns Hopkins Bloomberg School of Public Health, Baltimore, United States of America.

## Abstract

**Objective:**

To examine trends in, and projections of, the prevalence of anaemia in women of reproductive age in low- and middle-income countries at national and subpopulation levels.

**Methods:**

We used nationally representative data from repeated cross-sectional Demographic and Health Surveys (DHS) on 1 092 512 women of reproductive age (15–49 years) from 15 low- and middle-income countries. We defined anaemia as haemoglobin < 11 g/dL for pregnant women and < 12 g/dL for non-pregnant women. We analysed data using Bayesian linear regression analyses.

**Findings:**

During 2000–2018, the prevalence of anaemia in women of reproductive age decreased in nine countries, with the highest decrease in Malawi (−2.5%), and increased in six countries, with the highest increase in Burundi (10.9%). All countries are projected to have a prevalence of anaemia ≥ 15% in 2025, with the highest level in Burundi (66.8%). The prevalence of anaemia and projection of prevalence varied between and within countries. Women’s education, family wealth and place of residence had the highest impact on the current and projected prevalence rates of anaemia. Seven countries had a prevalence of anaemia ≥ 40%, which we defined as a severe public health problem, in the earliest and latest DHS and this prevalence is projected to persist in 2025.

**Conclusion:**

None of the 15 countries is likely to meet the global nutrition target of a 50% reduction in the prevalence of anaemia in women of reproductive age by 2025. Global and country leaders should reconsider nutrition policies and reallocate resources targeting countries and communities at risk.

## Résumé

**Objectif:**

Examiner les tendances et projections en ce qui concerne la prévalence de l'anémie chez les femmes en âge de procréer dans les pays à faible et moyen revenu, tant au niveau national qu'au sein des sous-populations.

**Méthodes:**

Nous avons utilisé des données représentatives à l'échelle nationale, extraites des enquêtes démographiques et sanitaires (EDS) transversales à passages répétés menées sur 1 092 512 femmes en âge de procréer (15–49 ans) originaires de 15 pays à faible et moyen revenu. Nous avons défini l'anémie comme correspondant à un taux d'hémoglobine < 11 g/dL pour les femmes enceintes, et < 12 g/dL pour celles qui ne le sont pas. Enfin, nous avons analysé ces données à l'aide d'un modèle de régression linéaire bayésienne.

**Résultats:**

Entre 2000 et 2018, la prévalence de l'anémie chez les femmes en âge de procréer a diminué dans neuf pays, surtout au Malawi (-2,5%). En revanche, elle a augmenté dans six pays, en particulier au Burundi (10,9%). Tous les pays devraient enregistrer une prévalence ≥ 15% en 2025, avec le taux le plus élevé au Burundi (66,8%). Des variations en termes de prévalence de l'anémie et de projections ont été observées entre les différents pays, mais aussi au sein d'un même pays. L'éducation des femmes, la situation financière de leur famille et leur lieu de résidence sont les éléments qui ont eu le plus d'impact sur les taux de prévalence actuels et prévisionnels. Sept pays ont enregistré une prévalence ≥ 40%, ce que nous considérons comme un grave problème de santé publique dans les premières et dernières EDS; cette prévalence devrait persister en 2025.

**Conclusion:**

Aucun des 15 pays étudiés ne devrait atteindre la cible mondiale de nutrition, qui consiste à réduire d'ici 2025 la prévalence de l'anémie de 50% chez les femmes en âge de procréer. Les dirigeants nationaux et mondiaux devraient repenser leurs politiques de nutrition et réaffecter les ressources, afin de privilégier les pays et les communautés à risque.

## Resumen

**Objetivo:**

Analizar las tendencias y las proyecciones de la prevalencia de la anemia en las mujeres en edad reproductiva de países de ingresos bajos y medios a nivel nacional y de subpoblación.

**Métodos:**

Se utilizaron los datos representativos a nivel nacional de repetidas Encuestas Demográficas y de Salud (EDS) transversales sobre 1 092 512 mujeres en edad reproductiva (15-49 años) de 15 países de ingresos bajos y medios. Se definió la anemia como una hemoglobina <11 g/dl para las embarazadas y <12 g/dl para las no embarazadas. Los datos se analizaron mediante un análisis de regresión lineal bayesiano.

**Resultados:**

Durante el periodo 2000-2018, la prevalencia de la anemia en las mujeres en edad reproductiva disminuyó en nueve países, con el mayor descenso en Malawi (-2,5 %), y aumentó en seis países, con el mayor incremento en Burundi (10,9 %). Se prevé que todos los países tengan una prevalencia de anemia ≥15 % en 2025, con el nivel más alto en Burundi (66,8 %). La prevalencia de la anemia y la proyección de la prevalencia variaron entre los países y dentro de ellos. La educación de las mujeres, la riqueza de la familia y el lugar de residencia fueron los factores que más influyeron en las tasas de prevalencia actual y proyectada de la anemia. Siete países tenían una prevalencia de anemia ≥40 %, que se definió como un problema grave de salud pública, en las primeras y últimas EDS, y se prevé que esta prevalencia persista en 2025.

**Conclusión:**

Es probable que ninguno de los 15 países cumpla la meta global de nutrición de reducir en un 50 % la prevalencia de la anemia en mujeres en edad reproductiva para 2025. Los líderes mundiales y nacionales deben replantear las políticas de nutrición y reasignar los recursos que se destinan a los países y comunidades en riesgo.

## ملخص

**الغرض:**

فحص اتجاهات وتوقعات انتشار فقر الدم لدى النساء في سن الإنجاب في الدول ذات الدخل المنخفض والدخل المتوسط على المستوى الوطني والمستوى السكاني الثانوي.

**الطريقة:**

قمنا باستخدام بيانات تمثيلية على الصعيد الوطني من المسوح السكانية والصحية المقطعية المستعرضة المتكررة على 1092512 امرأة في سن الإنجاب (من 15 إلى 49 سنة) من 15 دولة من الدول ذات الدخل المنخفض والدخل المتوسط. حددنا فقر الدم على أنه نسبة الهيموغلوبين الأقل من 11 جم/ديسيلتر للنساء الحوامل، وأقل من 12 جم/ديسيلتر للنساء غير الحوامل. كما قمنا بتحليل البيانات باستخدام تحليلات التحوف الخطي Bayesian.

**النتائج:**

خلال الفترة من 2000 إلى 2018، انخفض انتشار فقر الدم لدى النساء في سن الإنجاب في تسع دول، وكان أعلى انخفاض في ملاوي (-2.5%)، وزاد في ست دول، وكانت أعلى زيادة في بوروندي (10.9%). من المتوقع أن يكون معدل انتشار فقر الدم في جميع الدول ≥ 15% في عام 2025، مع أعلى مستوى في بوروندي (66.8%). تفاوت انتشار فقر الدم وتوقع انتشاره بين الدول وداخلها. كان لتعليم المرأة، وثروة الأسرة، ومكان الإقامة، أكبر تأثير على معدلات الانتشار الحالية والمتوقعة لفقر الدم. كان معدل انتشار فقر الدم في سبع دول ≥ 40%، وهو ما عرّفناه بأنه مشكلة صحية عامة خطيرة، في أقدم وأحدث المسوح السكانية الصحية (DHS)، ومن المتوقع أن يستمر هذا الانتشار في عام 2025.

**الاستنتاج:**

ليس من المحتمل أن تلبي أية دولة من الدول الخمسة عشر هدف التغذية العالمي المتمثل في خفض معدل انتشار فقر الدم لدى النساء في سن الإنجاب بنسبة 50% بحلول عام 2025. يجب على القادة العالميين وقادة الدول إعادة النظر في سياسات التغذية، وإعادة تخصيص الموارد التي تستهدف الدول والمجتمعات التي تواجه الخطر.

## 摘要

**目的:**

在全国和分组人口层面调查低收入和中等收入国家育龄妇女贫血患病率的趋势和预测。

**方法:**

我们使用来自 15 个低收入和中等收入国家的 1,092,512 名育龄妇女（15-49 岁）的反复横断面人口健康调查 (DHS) 中具有全国代表性的数据。我们将贫血定义为孕妇血红蛋白 < 11 g/dL，非孕妇的血红蛋白 < 12 g/dL。我们使用贝叶斯定理线性回归分析法对数据进行分析。

**结果:**

2000-2018 年期间，育龄妇女贫血患病率在九个国家下降，其中马拉维下降幅度最大 (-2.5%)，在六个国家上升，其中布隆迪上升幅度最大 (10.9%)。预计到 2025 年，所有国家的贫血患病率将达到³15%，其中布隆迪的患病率最高 (66.8%)。贫血患病率和患病率预测在各个国家之间和各个国家内部各不相同。妇女的教育、家庭财富和居住地对当前预计的贫血患病率影响最大。在最早和最新的 DHS 中，七个国家的贫血患病率为 ³40%，我们将其定义为严重的公共卫生问题，预计这种患病率将持续到 2025 年。

**结论:**

15 个国家均不可能在 2025 年实现育龄妇女贫血患病率降低 50% 的全球营养目标。全球和国家领导人应重新考虑营养政策，并对处于风险的地区和国家重新分配资源。

## Резюме

**Цель:**

Изучить тенденции и прогнозы распространенности анемии среди женщин репродуктивного возраста в странах с низким и средним уровнем дохода на национальном и субпопуляционном уровнях.

**Методы:**

Авторы использовали национальные репрезентативные данные из повторных перекрестных демографических и медико-санитарных обследований (DHS) 1 092 512 женщин репродуктивного возраста (15–49 лет) из 15 стран с низким и средним уровнем дохода. Авторы определили анемию как уровень гемоглобина < 11,0 г/дл для беременных женщин и < 12,0 г/дл для небеременных женщин. Авторы проанализировали данные с помощью анализа байесовской линейной регрессии.

**Результаты:**

В течение 2000–2018 гг. распространенность анемии среди женщин репродуктивного возраста снизилась в девяти странах, при этом наибольшее снижение произошло в Малави (–2,5%), и увеличилась в шести странах, при этом максимальное увеличение произошло в Бурунди (10,9%). Прогнозируется, что во всех странах распространенность анемии составит ≥ 15% в 2025 г. с самым высоким уровнем в Бурунди (66,8%). Распространенность анемии и прогноз распространенности различались как внутри стран, так и между странами. Наибольшее влияние на текущие и прогнозируемые показатели распространенности анемии оказали уровень образования женщин, благосостояние семьи и место жительства. В семи странах распространенность анемии составляла ≥ 40%, которую авторы в самом раннем и последнем DHS определили как серьезную проблему общественного здравоохранения. По прогнозам, эта распространенность сохранится в 2025 году.

**Вывод:**

Ни одна из 15 стран, вероятно, не достигнет глобальной цели в области питания, а именно снижения на 50% распространенности анемии среди женщин репродуктивного возраста к 2025 году. Мировые лидеры и руководства стран должны пересмотреть политику в области питания и перераспределить ресурсы, ориентируясь на страны и группы женщин, которые находятся в зоне риска.

## Introduction

Anaemia among women of reproductive age is a major public health challenge that leads to serious health consequences for mothers.^1^ Annually, more than 115 000 maternal deaths are attributed to anaemia worldwide.^2^ Globally, nearly two in every five pregnant women and one in every three non-pregnant women of reproductive age have anaemia globally.^3^ The prevalence of anaemia in women of reproductive age is highest in low- and middle-income countries,^3^^,^^4^ likely due to the interplay of dietary factors, nutrient deficiencies and increased incidence of infectious diseases such as malaria, human immunodeficiency virus infection and parasitic infestations.^4^

Anaemia in women of reproductive age has long-term consequences. Women with anaemia are at increased risk of adverse birth outcomes,^5^ including increased risk of maternal death and delivering a low-birth-weight baby or small-for-gestational-age baby.^2^^,^^6^^–^^8^ Anaemia in mothers is also associated with anaemia in their offspring^9^ which may continue during the life course.

In 2012, the Sixty-fifth World Health Assembly approved an action plan for improving maternal, infant and child nutrition, and set global nutrition targets that Member States approved and agreed to meet. One of the global nutrition targets is a 50% reduction in the prevalence of anaemia in women of reproductive age by 2025.^10^ To achieve this reduction, several strategies, such as improvement in dietary intake, food diversification, food fortification, and iron and folic acid supplementation for pregnant and lactating women, are being implemented globally.^3^^,^^11^^,^^12^ However, these strategies need to consider the local context so that appropriate interventions can be put in place, particularly for marginalized communities.

To measure the success of the action plans in achieving the global nutrition targets for anaemia, routine monitoring and evaluation of progress and projection of future directions are essential. The last time evaluation of progress was done was in 2011, before the global nutrition targets were set.^3^ Furthermore, to date, no projections have been made to identify which countries and populations within countries are unlikely to achieve the anaemia-related global nutrition targets. Projections of anaemia in women of reproductive age across different sociodemographic groups are central to identifying the key priority areas or groups (i.e. identifying the most disadvantaged groups to be covered by interventions) and reinforcing or reformulating policies to achieve country goals. However, few data exist on the prevalence of anaemia in various subgroups, which impedes the assessment of current strategies and design of effective planning for further actions.

Our study aimed to examine the trends in, and projections of, the prevalence of anaemia in women of reproductive age in low- and middle-income countries at national and subpopulation levels. We also reviewed the nutritional policies of the countries included in our study and their influence on reducing anaemia.

## Methods

### Data source

In this secondary analysis, we used data obtained from nationally representative repeated cross-sectional surveys conducted between 1995 and 2018 under the Demographic and Health Surveys (DHS) programme.^13^ The programme collects a range of health indicators and their sociodemographic determinants. The surveys generally apply a uniform procedure and use a multistage sampling technique. 

### Participants and data collection

We retrieved data on women of reproductive age (15–49 years) for whom the DHS provided consistent information on anaemia. The DHS followed standard procedures to collect data and measure haemoglobin, which was done by trained medical personnel, as previously described.^14^ Anaemia was determined using altitude-adjusted haemoglobin concentration and classified according to the World Health Organization (WHO).^15^ For a non-pregnant woman, anaemia was defined as haemoglobin level < 12 g/dL and for a pregnant woman, anaemia was defined as haemoglobin level < 11 g/dL.^15^ See data repository for the sample selection procedure.^16^

### Global anaemia target 

The global nutrition target for anaemia in women of reproductive age is a 50% decrease in the prevalence of anaemia from baseline (2012) by 2025.^17^ Globally, therefore, the prevalence of anaemia is expected to fall from 29% in 2012 to 15% by 2025.^17^ Hence, we calculated the probability of attaining a target prevalence of anaemia of ≤15% among women of reproductive age. In addition, we assessed the burden due to anaemia for each country and subgroup within countries during the earliest and latest DHS rounds and projected the burden in 2025. To assess the burden, we used WHO criteria to classify countries as follows: no public health problem if the prevalence of anaemia in women of reproductive age was < 5.0%; mild public health problem if the prevalence of anaemia was 5.0–19.9%; moderate public health problem if the prevalence of anaemia was 20.0–39.9%; and severe public health problem if the prevalence of anaemia was ≥40.0%.^18^

### Analyses

For trend analysis, we considered 15 countries that had data on anaemia in women of reproductive age for at least two DHS rounds with the latest survey conducted in 2016 or later. First, we estimated the weighted prevalence (as a proportion) of anaemia from the original survey data for all survey years of each country. We calculated the prevalence of anaemia at national and subgroup levels according to women’s place of residence, education, age and wealth quintile based on household assets by principal component analysis as previously described.^19^ We classified education into two categories: lower than secondary school (no schooling or primary school), and secondary school or higher. Similarly, we categorized age as 15 to 19 years (adolescents) and 20 to 49 years (adults), and place of residence as rural and urban. We categorized wealth quintiles as poorest (first quintile), poorer, middle, richer and richest (fifth quintile).

To examine trends and projections, we applied a Bayesian linear regression model that used a Markov chain Monte Carlo algorithm of multiple imputations for missing data to estimate the trends and projections of anaemia in women of reproductive age from 2000 to 2025^20^ (data repository).^16^ We used this approach as we were interested in calculating the probability of achieving the 2025 target. We performed a logit transformation of all proportions. We did all the calculations after this transformation and then transformed back to probabilities to ensure that the predicted probabilities lay between 0 and 1. We considered time as a covariate in each model. For each model, we discarded the first 5000 iterations as burn-in. We increased the number of iterations until convergence diagnosis was reached for the output. For each parameter, we reported 95% credible intervals (CrI) drawn from 30 000 samples from the respective posterior distributions. The intercept and covariate effect sizes were estimates using non-informative normal distribution priors with a mean 0 and very low precision (i.e. 0.0001). We performed sensitivity analyses by examining trends for some countries from 2005 or later whenever possible. We compared our estimates from regression models with those from the original microdata to validate our estimates (data repository).^16^ We restricted our analysis to the country level rather than the regional level for two reasons. First, some regions had very few countries and there was heterogeneity between survey years and second, we were interested in assessing progress across individual countries so that country-level programmes and policies could be implemented.

Finally, we quantified changes in the odds of anaemia among women of reproductive age over time by applying binary logistic regression analysis after adjusting for wealth quintile, place of residence, education and age of women, and controlling cluster variations.

Data were analysed in Stata, version 15.1 (StataCorp. LP, College Station, United States of America) and R, version 3.5.

### Ethical considerations

This study was an analysis of secondary data from DHS. Data are de-identified and publicly available through MEASURE DHS. Our study used this publicly available anonymous data. The DHS survey method and questionnaire were reviewed and approved by the ICF Institutional Review Board, and all participants gave informed consent. 

## Results

### Sample characteristics

We included 1 092 512 women aged 15 to 49 years, from 44 surveys in 15 countries (data repository).^16^ Overall, 17.8% (205 727/1 092 512) of the women were in the poorest wealth quintile, 64.9% (731 069/1 092 512) were rural dwellers and 47.8% (515 537/1 092 512) had lower than secondary-school education. (These numbers are unweighted and do not exactly match the weighted percentages.) All the fitted models for projection analysis achieved convergence. See the summary of potential scale reduction factor values in the data repository.^16^

### Trends in anaemia

**Fig. 1:**
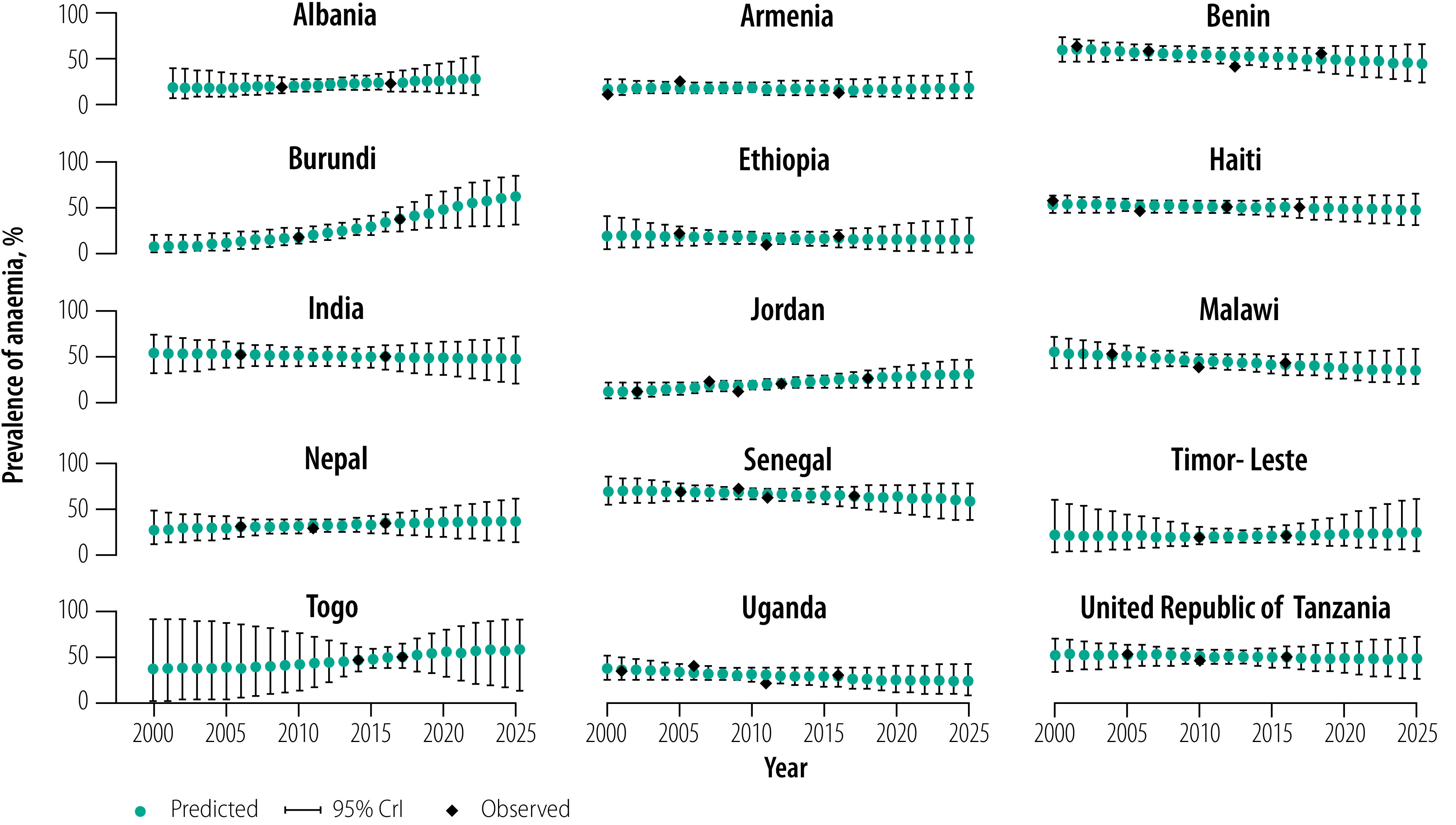
Observed and projected prevalence of anaemia in women of reproductive age in low- and middle-income countries, 2000–2025

**Fig. 2:**
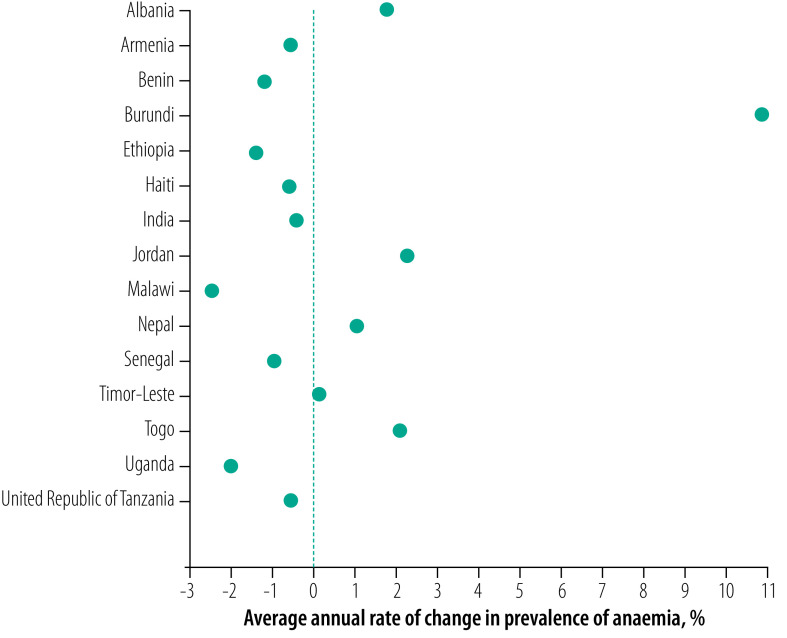
Average annual rate of change in the prevalence of anaemia in women of reproductive age in low- and middle-income countries, 2000–2018

Note: Average annual rate of change = ln[(rate in latest year/rate in earliest year)]/(rate in latest year – rate in earliest year).

Between 2000 and 2018, the prevalence of anaemia in women of reproductive age declined in nine of 15 countries (Fig. 1 and Fig. 2). The countries with the greatest reduction in the prevalence of anaemia were Malawi (−2.5%), Uganda (−2.0%) and Ethiopia (−1.4%; Fig. 2). However, the prevalence of anaemia increased in six countries with the highest increase in Burundi (10.9%) followed by Jordan (2.3%) and Togo (2.1%; Fig. 1 and Fig. 2). If the current trends continue, the prevalence of anaemia in 2025 is projected to be ≥15% in all 15 countries with the highest burden (prevalence) estimated for Burundi (66.8%; 95% CrI: 33.1 to 91.0), Togo (60.4%; 95% CrI: 12.9 to 96.4) and India (50.3%; 95% CrI: 22.7 to 76.2). Nearly half of the countries had a severe public health problem due to anaemia (i.e. prevalence ≥40%) in the earliest (seven countries) and latest (eight countries) DHS rounds and the problem is projected to remain severe until 2025 (data repository).^16^ No country had a greater than 50% probability of reducing anaemia to ≤15%, except Armenia, which we projected had a 53% probability of attaining this target (data repository).^16^

CrI: Credible interval.

**Fig. 3:**
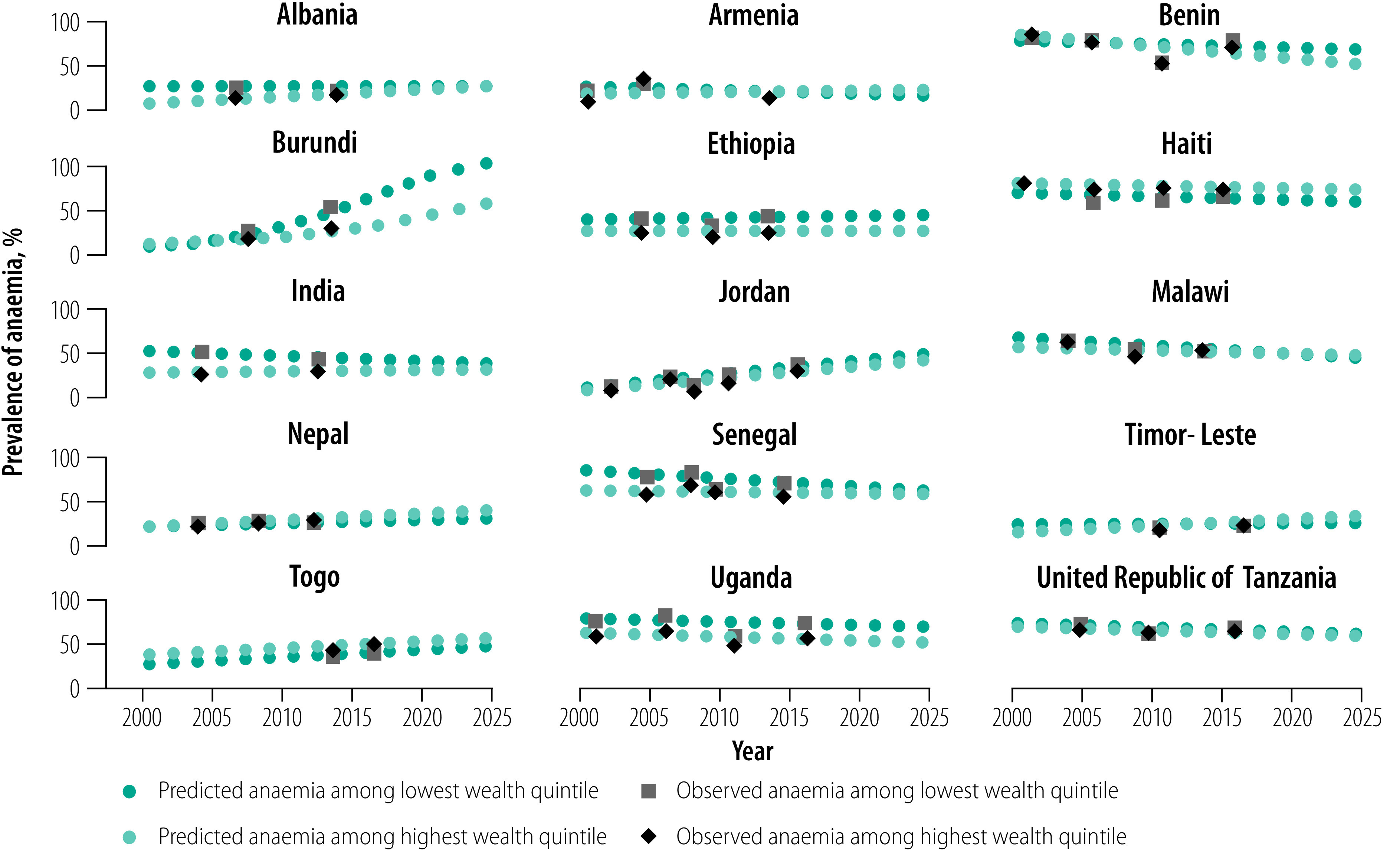
Trends in and projections of the prevalence of anaemia in women of reproductive age by wealth quintile in low- and middle-income countries, 2000–2025

Trends in the prevalence of anaemia varied across wealth quintiles (Fig. 3). Between 2000 and 2018, the prevalence of anaemia among the poorest women decreased in nine countries (Malawi, Armenia, Senegal, India, Haiti, United Republic of Tanzania, Uganda, Benin and Timor-Leste), with the largest decrease observed in Malawi (−2.5%); data repository.^16^ The other six countries saw an increase in the prevalence of anaemia in the poorest women, with the largest increase in Burundi (12.4%). Seven countries (Benin, Malawi, Uganda, United Republic of Tanzania, Ethiopia, Senegal and Haiti) saw a decrease in the prevalence of anaemia in the richest women and the remaining eight countries saw an increase (Fig. 3); Benin had the largest decrease (−1.8%) and Burundi had the highest increase (7.1%; data repository).^16^ Based on these trends, in 2025, the highest predicted prevalence of anaemia among the poorest women will be in Burundi (79.1%; 95% CrI: 48.7 to 95.7); among the richest women, the highest predicted prevalence will be in Togo (60.7%; 95% CrI: 10.0 to 96.7). In Armenia, the poorest women have a 67% probability of reaching a target prevalence of anaemia of ≤15%. In Ethiopia, the richest women have a 55% probability of meeting this target. In all other countries, women have a ≤50% chance of attaining the ≤15% target, irrespective of wealth (data repository).^16^

**Fig. 4:**
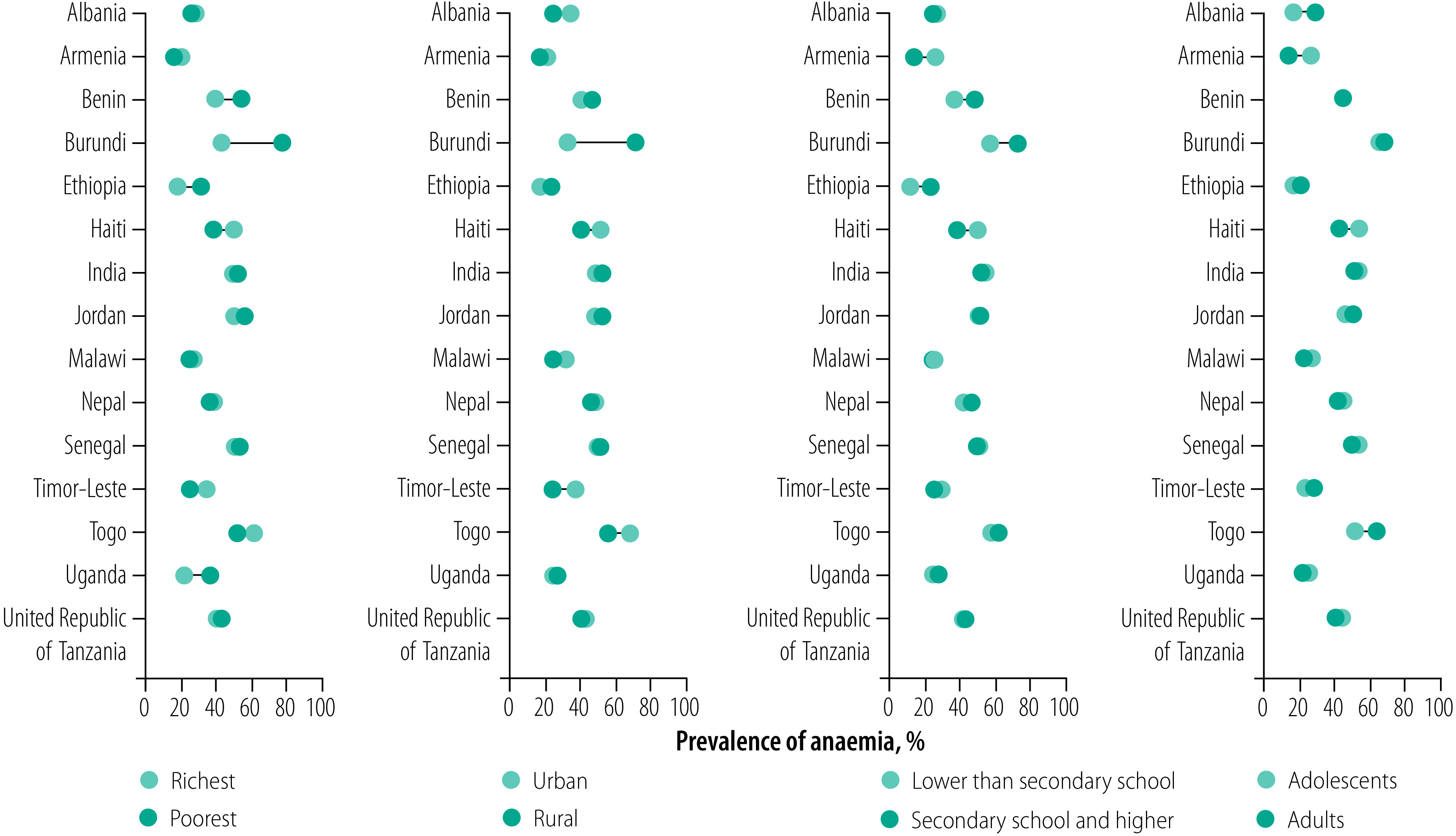
Predicted gaps in the prevalence of anaemia in women of reproductive age between categories of subgroups in low- and middle-income countries, 2025

Between 2000 and 2018, the prevalence of anaemia in women of reproductive age in rural areas declined in 11 of 15 countries (data repository),^16^ with the largest decline in Malawi (−2.8%; data repository).^16^ The prevalence of anaemia in women living in rural areas increased in four countries with the highest increase in Burundi (11.7%). In contrast, in seven countries the prevalence declined in women living in urban areas, with the highest decline in Benin (−1.6%). Of the eight countries that experienced an increase in anaemia in women in urban areas, Albania had the highest increase (4.6%). If the current trends persist, the prevalence of anaemia in women in 2025 is projected to be highest in rural areas in Burundi (70.9%; 95% CrI: 40.7 to 92.2) and in urban areas in Togo (66.2%; 95% CrI: 17.5 to 97.0). As with wealth-related trends, only Armenia has a > 50% probability (64%) of attaining a target of ≤15% prevalence who live in rural areas. In addition, only Ethiopia has a > 50% probability of attaining the target (64%) for women living in urban areas (data repository).^16^ The trends in the prevalence of anaemia also varied by women’s education and age (data repository).^16^ Furthermore, the projections indicate that while anaemia in women is decreasing in many countries, some countries will have large gaps in the prevalence across wealth, residence, education and age subgroups (Fig. 4). As with the subgroup projections, the public health burden of anaemia varied across subgroups (data repository).^16^

Findings of the sensitivity analysis were similar to the estimates from the standard analysis in most countries, apart from some variations in projected estimates over time (data repository).^16^

### Changes in risk of anaemia

**Fig. 5:**
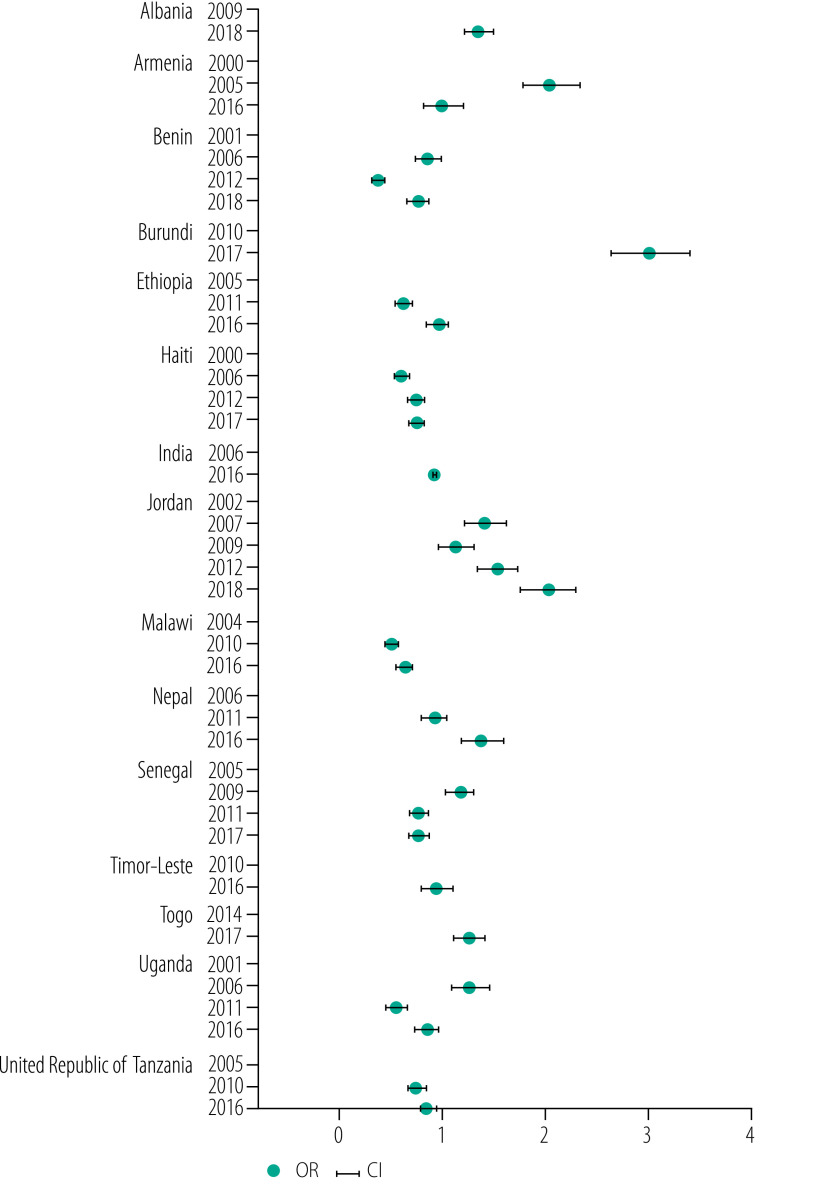
Adjusted odds ratio for change in the prevalence of anaemia over time among women of reproductive age in low- and middle-income countries, 2000–2018

CI: confidence interval; OR: odds ratio.

Note: Adjusted by wealth quintile, residence (urban versus rural), education and age.

We evaluated changes in the odds of anaemia in women of reproductive age over time in the 15 countries after adjusting for wealth quintile, residence, education and age. When the latest DHS data were compared with the earliest DHS data, the risk of anaemia in women had decreased significantly in seven countries and increased significantly in five countries (Fig. 5). Burundi had the highest increased risk of anaemia (adjusted odds ratio 3.01; 95% confidence interval: 2.66 to 3.40). The trends in the odds of the prevalence of anaemia over time were not stable across all countries, and some countries, e.g. Armenia, Ethiopia and Timor-Leste, showed no change in anaemia risk levels (Fig. 5).

## Discussion

The World Health Assembly target for reducing anaemia in women of reproductive age is an important target for evaluation of progress and projection of future directions. Our findings indicate a large disparity in the prevalence of anaemia in women of reproductive age between the 15 countries studied, which varied by place of residence, age, education and wealth. While nine countries saw a reduction in the prevalence of anaemia, no country is projected to reach a prevalence of ≤15% by 2025.

Our trend analysis shows consistent results with other studies.^3^ We identified considerable progress in reducing the prevalence of anaemia in women of reproductive age in some countries. However, except for Armenia, none of these countries has a ≥50% probability of reaching the target of ≤15% prevalence of anaemia by 2025 and 10 countries have a ≤10% probability. Hence, most of these countries will still have a severe public health problem due to anaemia in 2025. Our investigation of the prevalence gaps in anaemia across subpopulations shows that most of the countries projected to experience a high burden of anaemia in 2025 will also have larger gaps between subgroups. On the other hand, some countries will have smaller gaps in the prevalence of anaemia.

While the gaps in the prevalence of anaemia between subgroups are generally becoming smaller, such as gaps between the poorest and richest quintiles, the situation will be reversed in some countries, meaning that the better-off groups, such as the richer, urban and more educated women of reproductive age, may have a higher prevalence of anaemia in 2025. These findings show that inequalities in the prevalence of anaemia are not the only driver of this anaemia burden. Among the key factors driving the high burden of anaemia are genetic disorders, frequent illness due to infectious diseases, unavailability of food, low intake of nutrient-rich foods and poor health-care services.^17^^,^^21^ These factors are disproportionately distributed across different population subgroups. We recommend further studies to identify the population-specific key factors leading to increased or persistent high rates of anaemia in women of reproductive age so that the developmental activities can focus on tackling these factors. In addition, both advantaged and disadvantaged populations should be covered under the intervention strategies depending on the context and need, especially in countries where most of the population is at risk of anaemia irrespective of sociodemographic background.

Progress in reducing anaemia in women of reproductive age was uneven between countries and subgroups within countries during 2000–2018. Some countries, such as Albania, Armenia, Burundi, Jordan, Nepal, Timor-Leste and Togo, showed an increase in the prevalence of anaemia in the latest DHS assessment compared with the earliest assessment. Of these countries, Armenia and Togo are yet to update their nutrition policies following the Sixty-fifth World Health Assembly.^22^ In addition, India, Senegal and Uganda have not yet adopted updated policies,^22^ even though they have seen reductions in anaemia in women of reproductive age. Given that both increases and decreases in anaemia were seen regardless of updated policies, continuous anaemia surveillance is needed, policies need to be reassessed and appropriate actions put in place to combat this burden.

As anaemia in women of reproductive age is influenced by many factors, initiatives to tackle this problem need to be taken by many sectors such as government, private organizations and development partners. Appropriate interventions should be designed and implemented to reduce anaemia targeting the populations at risk. These interventions could include fortification of foods with nutrients, improving the quality of women’s diets, particularly women who are pregnant, and reducing morbidity due to infections. In addition, priority should be given to countries with a greater likelihood of a high anaemia burden and populations at risk of a higher prevalence of anaemia.

Most of the countries in our study have taken strategic initiatives to tackle anaemia. These initiatives include, but are not limited to, the food-assisted maternal and child health and nutrition programme (called Tubaramure) in Burundi,^23^ fortification of foods with micronutrients in Armenia,^24^ Burundi,^23^ Haiti,^25^ Malawi,^26^ Timor-Leste^27^ and Togo,^28^ and micronutrient supplementation in Ethiopia,^29^ India,^30^ Malawi,^26^ Nepal,^31^ Senegal,^32^ United Republic of Tanzania^33^ and Togo^28^ (programmes, interventions and strategies are available in the data repository).^16^ Some countries such as India have targeted adolescent girls through micronutrient supplementation programmes^30^ to eliminate anaemia before their adult motherhood stage. However, all countries need to revisit their national nutrition policies, adopt updated national nutrition policies and implement strategic actions in a multisectoral collaborative approach to meet the global nutrition target of reducing anaemia in women of reproductive age.

The main strength of our study is the use of population-based nationally representative samples covering both rural and urban areas and the analysis of population subgroups. Analysis of subgroups is particularly helpful to design interventions for groups at greatest risk of anaemia. The use of the same DHS method across countries allows cross-country comparison of the estimates. Despite this feature, our study has some limitations. Fewer data points created wider CrI for the projected estimates in some countries. However, CrI were smaller for countries with many data points. Wider CrI are normal for projection analysis, and calculation of realistic probability estimates are possible with wider CrI. Estimates drawn from authentic representative data collected from multiple sources may better predict the indicator with lower uncertainty. Finally, the projected estimates may be affected by the interrupted health services due to the current coronavirus disease 2019 (COVID-19) pandemic.^34^ The disruption to the economy, food supply, coverage of nutrition-specific interventions and health care during the COVID-19 pandemic may increase the burden of anaemia. However, progress on anaemia reduction may largely depend on the post-pandemic resilience of diet quality and health systems.

In conclusion, anaemia in women of reproductive age is high in most countries in our study with significant between- and within-country variations in trends and projections of this burden. These variations are expected to continue in 2025. No country is likely to achieve the global target of reducing anaemia unless the inequalities are minimized and effective interventions are implemented. The identification of countries and subpopulations provides an unparalleled opportunity for global and country leaders, policy-makers and programme managers to revisit strategies, reformulate policies and reallocate resources targeting the communities at risk.
